# Prevalence of Post-COVID conditions among Mexican COVID-19 survivors: a nationwide cross-sectional study

**DOI:** 10.1186/s12889-024-19274-3

**Published:** 2024-06-28

**Authors:** Yenisei Ramírez-Toscano, Leticia Torres-Ibarra, Martha Carnalla, Ana Basto-Abreu, Dèsirée Vidaña-Perez, M. Arantxa Colchero, Sergio Bautista-Arredondo, Sharon Saydah, Tonatiuh Barrientos-Gutiérrez

**Affiliations:** 1 Center for Population Health Research, National Institute of Public Health, Santa María Ahuacatitlán, Avenida Universidad 655, Cuernavaca, Morelos CP 62100 Mexico; 2 Department of Health Promotion Education and Behavior, Arnold School of Public Health, University of South Carolina, Columbia, SC USA; 3 Center for Health Systems Research, National Institute of Public Health, Cuernavaca, Morelos, Mexico; 4 Centers for Disease Control and Prevention, National Center for Immunization and Respiratory Diseases, Coronaviruses and Other Respiratory Viruses Division, Atlanta, GA USA

**Keywords:** COVID-19, Post-COVID condition, Long COVID, Epidemiology, Population-based study, Mexico

## Abstract

**Background:**

There are limited population-representative data that describe the potential burden of Post-COVID conditions (PCC) in Mexico. We estimated the prevalence of PCC overall and by sociodemographic characteristics among a representative sample of adults previously diagnosed with COVID-19 in Mexico. We additionally, characterized the PCC symptoms, and estimated the association between diagnosed type-2 diabetes and hypertension with PCC.

**Methods:**

We used data from the 2021 National Health and Nutrition Survey in Mexico, a nationally and regionally representative survey, from August 1st to October 31st, 2021. Using the WHO definition, we estimated the prevalence of PCC by sociodemographics and prevalence of PCC symptoms. We fit multivariable log-binomial regression models to estimate the associations.

**Results:**

The prevalence of PCC was 37.0%. The most common persistent symptoms were fatigue (56.8%), myalgia or arthralgia (47.5%), respiratory distress and dyspnea (42.7%), headache (34.0%), and cough (25.7%). The prevalence was higher in older people, women, and individuals with low socioeconomic status. There was no significant association between hypertension and PCC or diabetes and PCC prevalence.

**Conclusions:**

About one-third of the adult Mexican population who had COVID-19 in 2021 had Post-COVID conditions. Our population-based estimates can help assess potential priorities for PCC-related health services, which is critical in light of our weak health system and limited funding.

**Supplementary Information:**

The online version contains supplementary material available at 10.1186/s12889-024-19274-3.

## Background

Four years after the COVID-19 pandemic first impacted the world, the estimation of the long-lasting health effects of the COVID-19 has received less attention, particularly in low- and middle-income countries. For the general public, the term Long COVID has emerged to broadly describe the persistence of symptoms after first being infected with SARS-CoV-2, regardless of severity. In scientific communications, Post-COVID conditions (PCC) being the preferred term. PCC is a multi-organ condition that include pulmonary, hematologic, cardiovascular, neuropsychiatric, renal, endocrine, gastrointestinal/hepatobiliary, and dermatologic affectations [1]. Although there is no consensus about the definition of PCC, the World Health Organization (WHO) describes that post-COVID-19 condition is present if the persistence of symptoms last for 12 weeks or more since the acute infection [2]. Whilst the National Institute for Health and Care Excellence (NICE) recognized a definition that includes two categories, ongoing symptomatic COVID-19 (from 4 to 12 weeks) and Post-COVID-19 syndrome (more than 12 weeks), US Centers for Disease Control and Prevention (CDC) define it as symptoms 4 or more weeks after infection [3, 4].

To date, the lack of a standard case definition of PCC and population-representative surveys has led to a wide variety of estimates of the people potentially affected by PCC. In United Kingdom, an estimated 3.1% of the general population self-reported PCC four weeks after the first confirmed or suspected COVID-19 infection as of January 2023 [5]. In the United States, an estimated 6.0% of US adults currently have symptoms of PCC lasting 3 months or more after infection during the two-week study period ending June 2023 [6]. Recently a meta-analysis summarized the data available and estimated a pooled global prevalence of PCC among people diagnosed with COVID-19 of 43% [7]. However, nearly all evidence has been obtained in the US, Europe, and Asia and little is known of the COVID-19 long-term consequences in Latin America [8]. Despite the heterogeneity of the research studies, most of them identified a pattern of similar symptoms, among which fatigue, dyspnea, anxiety, myalgia or arthralgia, and concentration difficulties stand out [9, 10]. The understanding of symptoms of a new syndrome remains crucial to enhance recognition and proper management of the cases.

Several studies have looked at the potential association between comorbidities like diabetes and hypertension and PCC [11–14]. Some literature points at a bidirectional association [15, 16], for instance, while some studies show associations between pre-existing diabetes and post-acute sequelae of COVID-19 [11, 17], other studies show that diabetes should be considered as a post-acute sequelae of COVID-19 [18, 19]. For hypertension, few studies focused on the link between the presence of pre-existing hypertension and PCC [20, 21]. Since hypertension and diabetes are two highly prevalent diseases in Mexican adults (49.4% [22] and 15.7% [23], respectively), understanding the association of having these conditions and PCC is crucial to identify those patients who require additional needs in clinical care and public health efforts.

Using data from the 2021 National Health and Nutrition Survey in Mexico, we aimed to estimate the prevalence of PCC among adults previously diagnosed with COVID-19 according to sociodemographic characteristics as well as to describe the most persistent symptoms. We also aimed to estimate the association between diagnosed type-2 diabetes and hypertension with PCC. Since COVID-19 infections are still occurring around the world, our findings might help to anticipate the PCC burden and identify key groups at higher risk.

## Methods

### Study design and population

The 2021 National Health and Nutrition Survey (Ensanut) is a nationally representative survey conducted from August 2021 to October 2021. The Ensanut has a probabilistic, multistage, stratified, and clustered sampling design, representative at the national, regional, and rural/urban levels [24]. The Ensanut aimed to understand the effects of the pandemic on health, besides the frequency of health and nutrition conditions and their determinants. From 16,747 selected households a total of 12,060 households were surveyed, with 43,724 members, resulting in a participation rate of 72.0%. The head of the household answered a questionnaire applied by a trained interviewer that included sociodemographic and health status information about each household member. A detailed description of the 2021 Ensanut survey can be found on the Ensanut website [25].

**Fig. 1:**
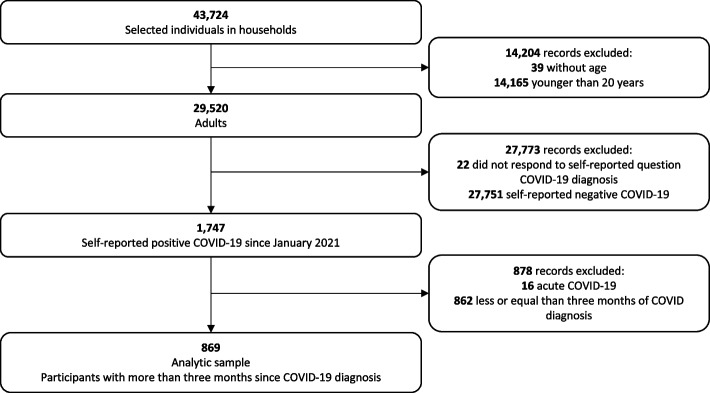
Flowchart of the study sample. Ensanut 2021

From 43,724 eligible participants in the household survey, 29,520 were adults aged 20 years and older. Our study population comprised subjects aged 20 years and older who according to the household member had been diagnosed with COVID-19 since January 1st, 2021 to the survey date which spanned from August 1st to October 31st, 2021 (*n* = 1,747). Subjects were considered to have had COVID-19 if they had been diagnosed with COVID-19 by a health-care professional either by symptoms or a positive SARS-CoV-2 test, according to the report of the head of the household. We excluded individuals who reported active COVID-19 at the time of the survey (*n* = 16), and those who had been diagnosed with COVID-19 within the last three months (*n* = 862). This last exclusion criterion is necessary to correctly classify individuals according into three mutually exclusive categories of the NICE definition, at least three months had to have elapsed since the diagnosis of COVID-19. That is, it could be the case that individuals with symptoms of less than 12 weeks could be classified as "ongoing symptomatic COVID-19", but could progress to "post-COVID-19 syndrome" if more than 3 months had elapsed between COVID-19 diagnosis and the survey date. After exclusions, 869 individuals remained in our analytical sample (Fig. 1).

This study followed the Strengthening the Reporting of Observational Studies in Epidemiology (STROBE) reporting guideline for cross-sectional studies [26].

### Outcome: Post-COVID conditions

We obtained the case definition as follows: First, we identified those participants previously diagnosed with COVID-19, using the question: “From January 2021 to date, was (YOU/NAME) diagnosed with COVID-19 by any health personnel?”; Then, the participants who responded affirmatively were asked about persistent symptoms, through the question: “After you were discharged or one month after your illness began, did you continue to present any of these symptoms/sequelae?” and interrogated the presence of the following symptoms: cough, fatigue, anxiety, depression, fever, insomnia, kidney complications, hyporexia, weight loss, headache, dizziness, myalgia or arthralgia, respiratory symptoms (includes respiratory distress and dyspnea), chest pain, vomit or diarrhea, anosmia, ageusia, trouble thinking or concentrating, others.

As we mentioned below, different organizations have established distinct case definitions for PCC, differing in terms of how long the symptoms continue. We follow the PCC definition proposed by the World Health Organization, as the persistence of one or more symptoms after three months of being diagnosed with COVID-19 [2]. We have adopted this definition because it was obtained through a protocol-based methodology that included patients, clinicians, and researchers from different economic and social contexts, and is representative of low- and middle-income countries.

### Exposure: Chronic diseases

The presence of diagnosed diabetes and hypertension was reported by the head of the household for all family members, using the question: “Has (YOU/NAME) ever been told by a physician that he/she have diabetes?”. The same question structure was used to assess the presence of hypertension. As the household questionnaire does not capture if the chronic diseases diagnosis were made before or after COVID-19 diagnosis, we did an analysis in a subsample that answered to the individual health questionnaire to know the percentage of those adults with a chronic disease diagnosis (diabetes and hypertension) before or after COVID-19 diagnosis. From this subsample (*n* = 408), only 5.6% and 20.2% of adults reported having a diabetes and a hypertension diagnosis, respectively, after being diagnosed with COVID-19 (see Supplementary Table 1).

### Sociodemographic characteristics

Individual level covariates included: sex, age (categorized into 20–39, 40–59, 60 years and older), education (elementary school or less, middle school, high school, graduate or higher). Affiliation to social security institutions was based in the response on survey question whether they are affiliated to a social security institution directly as part of their working status or as member of their family (categorized into with and without affiliation). Socioeconomic status was previously constructed using principal component analysis considering the household’ characteristics, ownership of a car, number of goods, number of electric appliances and services (running water) and then divided in tertiles (low, middle, high) [27]. Urbanization was defined based on the number of inhabitants in the locality of residence and was categorized as rural (< 2,500 inhabitants), urban (2,500–100,000 inhabitants) and metropolitan (> 100,000 inhabitants).

### Statistical analysis

The overall weighted prevalence of PCC with 95% confidence intervals (95% CIs) was estimated by all sociodemographic characteristics. We also estimated the weighted prevalence of PCC symptoms. To estimate the association between chronic diseases —diabetes and hypertension— and PCC, we fitted two separate multivariable log-binomial regression models adjusted by sex and age with people without PCC as reference. Covariates were selected using Directed Acyclic Graphs and variables were included from the minimal sufficient adjustment sets for estimating the total effect of chronic diseases on PCC. We chose the log-binomial regression approach due to our non-rare outcome (prevalence > 10%) and the model produces an unbiased estimate [28, 29]. The final models were checked for violations from the model assumptions. We reported adjusted prevalence ratios (aPR) with 95% CIs and P values.

We ran a secondary analysis exploring the prevalence of long-term effects of COVID-19 according to the NICE guidelines [3]. We created three categories “none”, “Ongoing symptomatic COVID-19” if the symptoms persisted between one month and three months; and “Post-COVID-19 syndrome” if the symptoms persisted more than three months. Then, we estimated the prevalence with a time frame of 4–12 weeks and > 12 weeks, and by all sociodemographic characteristics. We also estimated the prevalence of each symptom in each category of the two-time frames defined. We fitted multivariable log-binomial regression models to estimate the association between chronic diseases and each time frame.

All the estimators considered the complex survey design (weights, strata, clusters) using the svy command in STATA software package version 17 (Stata Corp, College Station, Texas, USA), and 2-sided P values < 0.05 were considered statistically significant. The analytic script (Do-file) for the statistical analysis is reported in the Supplementary Document 1.

### Ethics statement

The Ensanut 2021 study was reviewed and approved by the Institutional Review Board of the National Institute of Public Health of Mexico; written informed consent was obtained from participants.

## Results

In this study we included 869 adults who reported having had COVID-19 diagnosis between January 1st to October 31st 2021. Table 1 presents the prevalence and population estimates of PCC among people who reported having had COVID-19 by sociodemographic characteristics. Overall, an estimated 939,679 individuals experienced PCC (37.0%; 95%CI, 32.7–41.5) during 2021. Although no significant differences were observed by sociodemographic characteristics, women were more likely than men to report PCC (41.7% [95%CI, 36.8, 46.8] vs 31.4% [95%CI, 25.4, 38.1]). The prevalence of PCC was greater with increased age, from 33.4% (95% CI, 27.6–39.9) in the 20–39 age group to 42.5% (95% CI, 31.2–54.6) in the 60 and older age group. Also, we found higher prevalence of PCC in people living in rural areas (48.8%; 95% CI, 37.9–59.8), those without affiliation to social security (40.4%; 95% CI, 34.5–46.5), and with middle school education (42.0%; 95%CI, 34.7–49.6). We found that PCC prevalence decreased as socioeconomic level increased, from 49.9% (95% CI, 40.0–59.8) in the low level to 33.0% (95% CI, 26.2–40.5) in the high level. Regarding chronic diseases, individuals with diabetes, or hypertension were more likely to report PCC than individuals without these conditions, respectively. We estimated the prevalence of long-term effects of COVID-19 by two additional time frames (4–12 weeks and > 12 weeks) and by sociodemographic characteristics (see Supplementary Table 2).

**Table 1:** Prevalence of Post-COVID conditions in Mexican adults diagnosed with COVID-19 during 2021. Ensanut 2021

	**Adults diagnosed with COVID-19**		**Post-COVID conditions**	
	**Sample size unweighted No**	**Weighted No. (millions)**^**a**^	**%**	**95%CI**
**Total**	869	2.5	37.0	32.7, 41.5
Sex				
Male	381	1.2	31.4	25.4, 38.1
Female	488	1.4	41.7	36.8, 46.8
Age (years)				
20–39	338	1.1	33.4	27.6, 39.9
40–59	360	1.0	38.2	31.5, 45.4
60 and older	171	0.5	42.5	31.2, 54.6
Urbanization (population)				
Rural (< 2,500 inhabitants)	96	0.3	48.8	37.9, 59.8
Urban (2,500–100,000 inhabitants)	264	0.7	34.8	26.5, 44.1
Metropolitan (> 100,000 inhabitants)	509	1.6	36.0	30.6, 41.7
Social security				
With affiliation	470	1.4	34.4	28.5, 40.8
Without affiliation	399	1.1	40.4	34.5, 46.5
Socioeconomic level				
Low	162	0.4	49.9	40.0, 59.8
Medium	296	0.8	36.9	29.9, 44.6
High	411	1.3	33.0	26.2, 40.5
Education				
Elementary school	189	0.5	33.1	25.4, 41.8
Middle school	225	0.6	42.0	34.7, 49.6
High school	251	0.8	39.7	32.0, 48.1
University or higher	204	0.7	32.2	24.2, 41.3
Comorbidities				
Diabetes				
No	745	2.2	35.8	31.6, 40.2
Yes	124	0.4	43.7	30.5, 57.9
Hypertension				
No	689	2.1	34.5	30.1, 39.1
Yes	180	0.5	47.7	37.4, 58.2

*Abbreviations*: *95*% *CI* 95% confidence interval

^a ^Weighted No. (millions) represents the expanded sample size of adults diagnosed with COVID-19

**Fig. 2:**
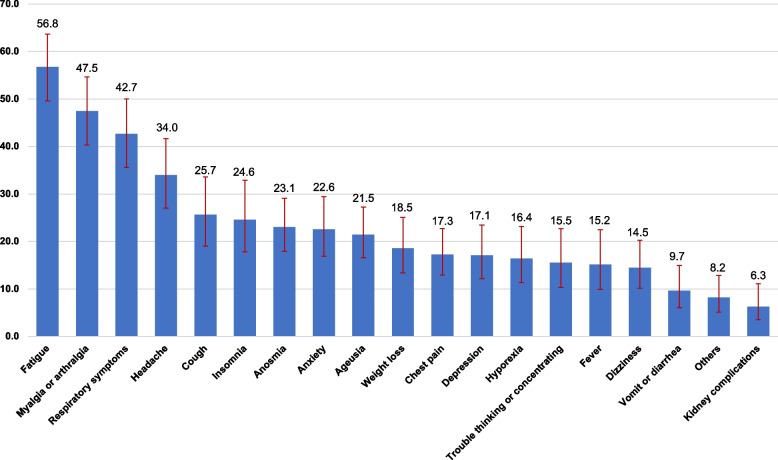
Prevalence of Post-COVID conditions symptoms in Mexican adults diagnosed with COVID-19 in 2021. Ensanut 2021 Respiratory symptoms include respiratory distress and dyspnea

Figure 2 shows the prevalence of individual symptoms of PCC. Among individuals with PCC the most common symptoms were fatigue (56.8%; 95% CI, 49.6–63.7), myalgia or arthralgia (47.5%; 95%CI, 40.3–54.7), respiratory symptoms (includes respiratory distress and dyspnea) (42.7%; 95%CI, 35.6–50.1), headache (34.0%; 95% CI, 27.1–41.7), and cough (25.7%; 95% CI, 19.1–33.6). We estimated the prevalence of individual symptoms by two-time frames of the long-term effects of COVID-19 according to NICE guidelines (4–12 weeks and > 12 weeks) (see Supplementary Fig. 1).

Table 2 shows the results from multivariable analysis on the association between diabetes and hypertension with PCC. There was no significant association between hypertension and PCC (PR = 1.29; 95%CI, 0.95–1.76) or diabetes and PCC prevalence (PR = 1.11; 95% CI, 0.84–1.47). Lastly, the results from secondary analyses that assessed the association between chronic diseases and the long-term effects of COVID-19 by two-time frames (4–12 weeks and > 12 weeks) showed similar results to the main models (see Supplementary Table 3 and Supplementary Table 4).

**Table 2:** Association between diabetes and hypertension with Post-COVID conditions in Mexican adults diagnosed with COVID-19 in 2021

	**Post-COVID conditions**^**a**^		
	**Yes vs no**		
	**Prevalence ratio**	**95% CI**	***P* value**
**Diabetes**^**b**^			
** No**	1 [Reference]	NA	NA
** Yes**	1.11	0.84, 1.47	0.452
**Hypertension**^**b**^			
** No**	1 [Reference]	NA	NA
** Yes**	1.29	0.95, 1.76	0.098

*Abbreviations*: *95*% *CI* 95% Confidence Interval, *NA* not applicable

Number of observations = 869

^a^Log-binomial regression models adjusted for sex and age. Participants without Post-COVID conditions was used as reference

^b^Chronic diseases were reported by the head of the household for all family members

## Discussion

Our findings suggest that 37.0% of the adult population in Mexico reported persistence of any symptoms 12 weeks after a COVID-19 diagnosis between January 2021 and October 2021. The most common persistent symptom among individuals with PCC for > 12 weeks was fatigue, followed by myalgia or arthralgia, respiratory symptoms (includes respiratory distress and dyspnea), headache, and cough. We found that people with physician-diagnosed hypertension might had a higher prevalence of PCC in comparison with people who did not report having the disease, although this association was not statistically significant.

The PCC prevalence in our study was similar to a previous meta-analysis of different regions of the world [7, 30–32]. The global estimated pooled prevalence of PCC by number of days after COVID-19 diagnosis was 37%, 25%, 32% and 49% for 30, 60, 90, and 120 days, respectively [7]. Another pooled estimates of PCC, mainly from high-income countries was 63.2%, 71.9% and 45.9% at 30, 60 or ≥ 90 days after COVID-19 onset [32]. In Mexico, a study in 2020 reported a prevalence of COVID-19 sequelae of 15.8%, but this was based on a population that tested seropositive for SARS-CoV-2 without information on the type and duration of symptoms [33].

Our results on most prevalent PCC symptoms are similar to other countries. A meta-analysis reported that the most common symptoms between 3 and < 6 months were fatigue, dyspnea, sleep disorders, and concentration difficulty [10]. Another systematic review and meta-analysis reported that the most prevalent symptoms at one-year follow-up were fatigue/weakness (28%), dyspnea (18%), arthromyalgia (26%), depression (23%), anxiety (22%), and memory loss (19%), concentration difficulties (18%), and insomnia (12%) [9]. Other studies also reported fatigue as the prevalent symptom in individuals with Post-COVID [1, 7, 30–32, 34, 35], similar to our results. Something that stands out is that the presence of these symptoms can lead to a loss of productivity and adversely affect the employment and the household finances of people who suffer from it. Indeed, a recent study reported that the presence of any cognitive symptoms among those with PCC, reduce the likelihood of working fulltime [36]. From the health system perspective, the recognition of symptoms may help healthcare providers and planners to develop strategies to ameliorate such symptoms and facilitate functional recovery.

In our study we found that individuals with diagnosed hypertension had a higher prevalence of PCC, although the association was not significant, perhaps due to factors described below in the limitations. For hypertension, prior studies found that pre-existing hypertension was a predictor for PCC in COVID-19 hospitalized patients 4 months after discharge [20], and 12 months after discharged [21]. Hypertension is known as a cause of endothelial dysfunction [37] and a higher risk of COVID-19 severe outcomes through this mechanism [38]; hence, the higher PCC prevalence could be a consequence of a higher COVID-19 severe outcomes. For diabetes, a multicenter case control study in Madrid, also found no association between pre-existing type-2 diabetes and PCC, in line with our study [39].

Interestingly, we observed that individuals in the low socioeconomic level had a higher prevalence of PCC than those in the high socioeconomic level (49.9% vs 33.0%). Evidence from the UK has shown similar results. A community-based longitudinal survey of COVID-19 in UK shows that participants in the most socioeconomically deprived deciles had a 45% higher risk of PCC [40]. Another study showed an increased risk of PCC in socioeconomically deprived participants [41]. A study from Brazil found that lower socioeconomic position was associated with increased post-COVID syndrome, specifically with dyspnea, increased fatigue, and worse functional status symptoms [42]. To date, there is no clear explanation for the role of socioeconomic inequalities in PCC. However, some hypotheses suggest that poor working, housing conditions and unhealthier lifestyle behavior among most deprived populations can lead to a higher likelihood to develop PCC [40, 43] Likewise, it would also be interesting to explore how the difficulties that people with lower socioeconomic status frequently encounter in accessing healthcare and taking care of themselves due to COVID-19 contribute to the rise in PCC in this subgroup.

Our study has some important limitations. First, the head of the household reported the presence of COVID-19 symptoms and their duration, as well as the diagnosis of chronic diseases of each house member, which could lead to both exposure and outcome misclassification. We quantify the validity of the chronic diseases reporting by the head of the household using the sensitivity and specificity of these data as compared with the self-reported of chronic diseases of a subsample of our population; for diabetes diagnosis we found a sensitivity of 94.4% and specificity of 97.7%, for hypertension the sensitivity was 83.1% and a specificity of 96.6% (see Supplementary Table 5, Supplementary Table 6, and Supplementary Table 7). However, we did not conduct a sensitivity analysis comparing data in which the diagnosis was confirmed by a positive SARS-CoV-2 test with symptomatic diagnosis by health care workers for two reasons: both the CDC and WHO have stated that a SARS-CoV-2 test (PCR, antigen, or antibody) is not required to establish a PCC diagnosis [2, 4]. More importantly, because we wanted to assess the prevalence of PCC and its symptoms in a general population, limiting our sample to those with a confirmed diagnosis confirmed by a positive SARS-CoV-2 test could result in selection bias, as only 15.1% of the population reported testing for SARS-CoV-2, according to the Ensanut 2021 report [27]. As a result, the population that sought and received COVID-19 testing may differ in terms of sociodemographics.

Second, the survey collected information about the last COVID-19 infection from January 2021 to the time of the survey (October 2021), hence, we are not capturing the cumulative number of cases of PCC originated from prior infections. Additionally, we are excluding respondents whose most recent SARS-CoV-2 infection was within the past three months to have enough time to meet the PCC definition (> 12 weeks). However, some of these respondents may have had PCC from an earlier infection, which would result in an underestimation of the current prevalence of PCC. Third, our analysis is based on those people who reported having had COVID-19 diagnosed by a health-care professional either by symptoms or a positive SARS-CoV-2 test. This would underestimate the number of people infected with SARS-CoV-2 who did not seek medical attention, both asymptomatic and symptomatic, resulting in a possible selection bias that yield to an overestimation of the PCC prevalence. Fourth, given the cross-sectional nature of the study, we cannot establish a causal association between chronic diseases and PCC. Also, we were not able to distinguish whether diabetes and hypertension diagnoses preceded COVID-19 diagnosis or followed it; however, in a subsample, the small proportion of adults reported having a diabetes diagnosis (5.6%) and a hypertension diagnosis (20.2%) after being diagnosed with COVID-19 might no impact on the estimates of our models (see Supplementary Table 1). Additionally, chronic diseases only considered diagnosed conditions, and a large proportion of the population with diabetes and hypertension are unaware of their status [22, 23], which represents an important source of misclassification of true exposure. In this case, we expect that this misclassification is non-differential and then biasing the estimates of the associations towards the null. Another limitation is regarding residual confounding since we cannot rule out confounding by unmeasured variables. Despite limitations, this study fills an important gap for the international and Mexican communities, as the results provide assessment of the burden of PCC and across different sociodemographic factors that could help to understand the public health burden and clinical needs of PCC.

## Conclusion

In summary, we found that 4 out of 10 participants who were diagnosed with COVID-19 from January 2021 to October 2021, experienced persistent symptoms 12 weeks after COVID-19 diagnosis. Beyond COVID-19 prevention, it is important that public health interventions also recognize PCC as a priority issue, from clinical identification to the provision of resources for care. Interventions to prevent COVID-19 infection must address inequalities in PCC by age, sex, and socioeconomic status. Our population-based estimates can help assess potential priorities for PCC-related health services, which is critical in light of our weak health system and limited funding.

## Disclaimer

The findings and conclusions in this report are those of the author(s) and do not necessarily represent the official position of the Centers for Disease Control and Prevention (CDC).

### Supplementary Information

Supplementary Material 1.

## Data Availability

This is the secondary analysis of publicly available data, which can be accessed by anyone at: https://ensanut.insp.mx/encuestas/ensanutcontinua2021/index.php.
