# Supplementary material for: Prevalence of Post-COVID conditions among Mexican COVID-19 survivors: a nationwide cross-sectional study
Source: BMC Public Health. 2024 Jun 28;24:1729. doi: 10.1186/s12889-024-19274-3 (PMC11212168; doi:10.1186/s12889-024-19274-3)
Supplement: Supplementary file 1 — Supplementary Material 1. [file 12889_2024_19274_MOESM1_ESM.docx]

**Supplemental Online Content**

**Supplementary Table 1.** Frequency of reporting a diagnosis of diabetes and hypertension among a subsample who answered the individual health questionnaire in Ensanut 2021.

**Supplementary Document 1.** Script Post-COVID Conditions analysis.

**Supplementary Table 2.** Prevalence of Long-term effects of COVID-19 in Mexican adults diagnosed with COVID-19 in 2021. Ensanut 2021.

**Supplementary Figure 1.** Prevalence of symptoms by Long-term effects of COVID-19 duration in Mexican adults diagnosed with COVID-19 in 2021. Ensanut 2021.

**Supplementary Table 3.** Association between diabetes and hypertension with Ongoing symptomatic COVID-19 (4-12 weeks) in Mexican adults diagnosed with COVID-19 in 2021. Ensanut 2021.

**Supplementary Table 4.** Association between diabetes and hypertension with Post-COVID-19 syndrome (>12 weeks) in Mexican adults diagnosed with COVID-19 in 2021. Ensanut 2021.

**Supplementary Table 5.** Concept of the Sensitivity and Specificity screening examinations.

**Supplementary Table 6.** Sensitivity and Specificity of diabetes diagnosis. Ensanut 2021.

**Supplementary Table 7.** Sensitivity and Specificity of hypertension diagnosis. Ensanut 2021.

|  | **Supplementary Table 1. Frequency of reporting a diagnosis of diabetes and hypertension among a subsample who answered the individual health questionnaire in Ensanut 2021^a^** | | | |
| --- | --- | --- | --- | --- |
|  | |  | **n** | **%** |
| **Diabetes diagnosis (overall)** | |  | **54** |  |
| Diabetes before COVID-19 | |  | 51/54 | 94.4 |
| Diabetes after COVID-19 | |  | 3/54 | 5.6 |
| **Hypertension diagnosis (overall)** | |  | **89** |  |
| Hypertension before COVID-19 | |  | 71/89 | 79.8 |
| Hypertension after COVID-19 | |  | 18/89 | 20.2 |
| ^a^ Only a subsample of 408 individuals who had COVID-19 answered an individual health questionnaire. From this subsample, 54 adults self-reported having type-2 diabetes, and 89 self-reported having a diagnosis of hypertension. | | | | |

**Supplementary Document 1. Script Post-COVID Conditions analysis.**

*==============================================================================*

///// POST-COVID CONDITIONS /////

* Datasets : Ensanut Continua 2021: Home/Members/COVID

* Web page : https://ensanut.insp.mx/encuestas/ensanutcontinua2021/descargas.php

* Level : Individual level

* Author : Team Post-COVID conditions INSP

*==============================================================================*

*** Not to pause or display the more message

set more off

*==============================================================================*

///// ENSANUT CONTINUA 2021 /////

*==============================================================================*

*------------------------------------------------------------------------------*

///// HOME/MEMBERS ENSANUT 2021 DATASET /////

*------------------------------------------------------------------------------*

*** Create directories

* Use "global" command to keep the original dataset and work dataset in folders

gl data="/Users/Hogar";

gl data2="/Users/Adultos";

gl bases="/Users/BD_trabajo";

*** Open Adults dataset

use "$data2/ensadul2021_entrega_w_15_12_2021.dta", clear

* Keep interest variables

keep ponde_f estrato est_sel region indice1 nseF nse5F FOLIO_I FOLIO_INT edad sexo fech_nac fecha_ini fecha_fin a0101 a0301 a0401 a0302 a0402m a0402a

* Rename variables

rename ponde_f ponde_f_a

rename estrato estrato_a

rename est_sel est_sel_a

rename region region_a

rename indice1 indice1_a

rename nseF nseF_a

rename nse5F nse5F_a

rename fecha_ini fecha_ini_a

rename fecha_fin fecha_fin_a

* Sort dataset by FOLIO

sort FOLIO_I FOLIO_INT

* Merge Adults dataset with Household members dataset

merge 1:1 FOLIO_INT using "$data/integrantes_ensanut2021_w_12_01_2022.dta"

rename _merge merge_adul_int

tab merge_adul_int

* Matching result from |

* merge | Freq. Percent Cum.

*------------------------+-----------------------------------

* Using only (2) | 30,322 69.35 69.35

* Matched (3) | 13,402 30.65 100.00

*------------------------+-----------------------------------

* Total | 43,724 100.00

sort FOLIO_I FOLIO_INT

* Merge Adults-Household members dataset with Socioeconomic status dataset

merge m:1 FOLIO_I using "$data/ENSANUT2021_NSE.dta"

rename _merge merge_adul_int_nse

tab merge_adul_int_nse

* Matching result from |

* merge | Freq. Percent Cum.

*------------------------+-----------------------------------

* Matched (3) | 43,724 100.00 100.00

*------------------------+-----------------------------------

* Total | 43,724 100.00

sort FOLIO_I FOLIO_INT

merge m:1 FOLIO_I using "$data/hogar_ensanut2021_w_14_12_2021.dta", keepusing(fecha_ini fecha_fin)

rename _merge merge_adul_int_nse_hog

tab merge_adul_int_nse_hog

* Matching result from |

* merge | Freq. Percent Cum.

*------------------------+-----------------------------------

* Matched (3) | 43,724 100.00 100.00

*------------------------+-----------------------------------

* Total | 43,724 100.00

unique FOLIO_I

*Number of unique values of FOLIO_I is 12619

*Number of records is 43724

unique FOLIO_INT

*Number of unique values of FOLIO_INT is 43724

*Number of records is 43724

*** Generate new variables

* Date of interview

* STEP 1: We generated a new date variable with a format that stata recognizes

gen date_interv=date(fecha_ini,"DMY")

gen month_interv=month(date_interv)

* Education level

gen edu_4=.

*(43,724 missing values generated)

replace edu_4=0 if h0317a==0 | h0317a==1 | h0317a==2

replace edu_4=1 if h0317a==3

replace edu_4=2 if h0317a==4 | h0317a==5 | h0317a==6 | h0317a==7 | h0317a==8

replace edu_4=3 if h0317a==9 | h0317a==10 | h0317a==11 | h0317a==12

label define edu_4 0 "Elementary or less" 1 "Middle school" 2 "High school" 3 "University or higher"

label value edu_4 edu_4

tab edu_4, miss

label var edu_4 "Education level"

* Sex

gen sex=.

replace sex=0 if h0302==1

replace sex=1 if h0302==2

label define sex 0 "Man" 1 "Woman"

label value sex sex

tab sex, miss

label var sex "Sex"

* Age

gen age_cat=.

replace age_cat=0 if h0303<10

replace age_cat=1 if h0303>=10 & h0303<20

replace age_cat=2 if h0303>=20 & h0303<40

replace age_cat=3 if h0303>=40 & h0303<60

replace age_cat=4 if h0303>=60 & h0303<999

label define age_cat 0 "0-9" 1 "10-19" 2 "20-39" 3 "40-59" 4 "60 and higher"

label value age_cat age_cat

tab age_cat, miss

label var age_cat "Age categorical"

* Syntoms PCC (>4 weeks)

* .

*1= a Tos

*2= b Fatiga

*3= c Ansiedad

*4= d Depresión

*5= e Fiebre

*6= f Dificultad para dormir

*7= g Complicaciones en el riñón

*8= h Falta de hambre.

*9= i Pérdida de peso

*10= j Dolor de cabeza

*11= k Mareo

*12= l Dolores en músculos o articulaciones

*13= m Dificultad para respirar

*14= n Falta de aire

*15= o Dolor en el pecho

*16= p Vómito o diarrea

*17= q Pérdida o disminución del olfato

*18= r Pérdida o disminución del gusto

*19= s Dificultad para pensar o concentrarse

*20= t Otro (especifique).

*21= u No tuve síntomas / secuelas

*22= x Aún no le han dado de alta/ no ha pasado un mes

*23= y NS/NR

global list_sin = "H1213A H1213B H1213C H1213D H1213E1 H1213F H1213G H1213H H1213I H1213J H1213K H1213L H1213M H1213N H1213O H1213P H1213Q H1213R H1213S H1213T"

br $list_sin

gen cough=0

foreach var in $list_sin {

replace cough=1 if (`var'=="A")

}

*

gen fatigue=0

foreach var in $list_sin {

replace fatigue=1 if (`var'=="B")

}

*

gen anxiety=0

foreach var in $list_sin {

replace anxiety=1 if (`var'=="C")

}

*

gen depre=0

foreach var in $list_sin {

replace depre=1 if (`var'=="D")

}

*

gen fever=0

foreach var in $list_sin {

replace fever=1 if (`var'=="E")

}

*

gen insomnia=0

foreach var in $list_sin {

replace insomnia=1 if (`var'=="F")

}

*

gen renal=0

foreach var in $list_sin {

replace renal=1 if (`var'=="G")

}

*

gen hyporexia=0

foreach var in $list_sin {

replace hyporexia=1 if (`var'=="H")

}

*

gen weightloss=0

foreach var in $list_sin {

replace weightloss=1 if (`var'=="I")

}

*

gen headache=0

foreach var in $list_sin {

replace headache=1 if (`var'=="J")

}

*

gen dizziness=0

foreach var in $list_sin {

replace dizziness=1 if (`var'=="K")

}

*

gen myalgia_arthralgia=0

foreach var in $list_sin {

replace myalgia_arthralgia=1 if (`var'=="L")

}

*

gen dyspnoea=0

foreach var in $list_sin {

replace dyspnoea=1 if (`var'=="M" | `var'=="N")

}

*

gen chest_pain=0

foreach var in $list_sin {

replace chest_pain=1 if (`var'=="O")

}

*

gen vomit_diarrhea=0

foreach var in $list_sin {

replace vomit_diarrhea=1 if (`var'=="P")

}

*

gen anosmia=0

foreach var in $list_sin {

replace anosmia=1 if (`var'=="Q")

}

*

gen ageusia=0

foreach var in $list_sin {

replace ageusia=1 if (`var'=="R")

}

*

gen trouble_concen=0

foreach var in $list_sin {

replace trouble_concen=1 if (`var'=="S")

}

*

gen other=0

foreach var in $list_sin {

replace other=1 if (`var'=="T")

}

*

gen asymptomatic=0

foreach var in $list_sin {

replace asymptomatic=1 if (`var'=="U")

}

*

gen less_month=0

foreach var in $list_sin {

replace less_month=1 if (`var'=="X")

}

*

* Other symptoms

gen other_symptoms=h1213e

replace other_symptoms="." if other_symptoms=="AGOTAMIENTO" | other_symptoms=="Mucho AGOTAMIENTO" | other_symptoms=="CANSANCIO" | other_symptoms=="CANSANCIO Y SUDABA MUCHO" | other_symptoms== "CANSANSIO" | other_symptoms=="Cansancio" | other_symptoms=="SUEÑO"

replace other_symptoms="." if other_symptoms=="ARDOR EN LA ESPALDA"| other_symptoms=="DOLOR DE ESPALDA"| other_symptoms=="DOLOR DE ESPALDA"| other_symptoms=="DOLOR DE ESPALDA"| other_symptoms=="PERDIDA DE CABELLO Y DOLOR DE ESPALDA"| other_symptoms=="DUEKEN LAS PIERNAS"

replace other_symptoms="." if other_symptoms=="AUN NO LO DAN DE ALTA"| other_symptoms=="AUN NO LA DAN DE ALTA"| other_symptoms=="AUN ESTA ENFERMA NO HA SALIDO DEL COVID"| other_symptoms=="NO LO HAN DADO DE ALTA APENAS EN SEPTIEMBRE"| other_symptoms=="TODAVIA NO LA DAN DE ALTA SIGUE EN CUARENTENA"| other_symptoms=="DOS DIAS POSITIVO"| other_symptoms=="SIGUE ENFERMO"

replace other_symptoms="." if other_symptoms=="BAJO DE PESO"| other_symptoms=="PERDIÓ PESO"

replace other_symptoms="." if other_symptoms=="EMOCIONALES"

replace other_symptoms="." if other_symptoms=="DIARREA"

replace other_symptoms="." if other_symptoms=="PERDIDA DE APETITO"

*

gen others=0

replace others=1 if other==1

replace others=2 if other_symptoms=="."

replace others=0 if others==2

*

replace fatigue=1 if h1213e=="AGOTAMIENTO" | h1213e=="Mucho AGOTAMIENTO" | h1213e=="CANSANCIO" | h1213e=="CANSANCIO Y SUDABA MUCHO" | h1213e== "CANSANSIO" | h1213e=="Cansancio" | h1213e=="SUEÑO"

*(11 real changes made)

replace myalgia_arthralgia=1 if h1213e=="ARDOR EN LA ESPALDA"| h1213e=="DOLOR DE ESPALDA"| h1213e=="DOLOR DE ESPALDA"| h1213e=="DOLOR DE ESPALDA"| h1213e=="PERDIDA DE CABELLO Y DOLOR DE ESPALDA"| h1213e=="DUEKEN LAS PIERNAS"

*(6 real changes made)

replace less_month=1 if h1213e=="AUN NO LO DAN DE ALTA"| h1213e=="AUN NO LA DAN DE ALTA"| h1213e=="AUN ESTA ENFERMA NO HA SALIDO DEL COVID"| h1213e=="NO LO HAN DADO DE ALTA APENAS EN SEPTIEMBRE"| h1213e=="TODAVIA NO LA DAN DE ALTA SIGUE EN CUARENTENA"| h1213e=="DOS DIAS POSITIVO"| h1213e=="SIGUE ENFERMO"

*(7 real changes made)

replace weightloss=1 if h1213e=="BAJO DE PESO"| h1213e=="PERDIÓ PESO"

*(2 real changes made)

replace depre=1 if h1213e=="EMOCIONALES"

*(1 real change made)

replace vomit_diarrhea=1 if h1213e=="DIARREA"

*(4 real changes made)

replace hyporexia=1 if h1213e=="PERDIDA DE APETITO"

*(1 real change made)

**Agregar a "fatigue"

*AGOTAMIENTO

*Mucho AGOTAMIENTO

*CANSANCIO

*CANSANCIO Y SUDABA MUCHO

*CANSANSIO

*Cansancio

*SUEÑO

**Agregar a "myalgia_arthralgia"

*ARDOR EN LA ESPALDA

*DOLOR DE ESPALDA

*DOLOR DE ESPALDA

*DOLOR DE ESPALDA

*PERDIDA DE CABELLO Y DOLOR DE ESPALDA

*DUEKEN LAS PIERNAS

**Agregar a "less_month"

*AUN NO LO DAN DE ALTA

*AUN NO LA DAN DE ALTA

*AUN ESTA ENFERMA NO HA SALIDO DEL COVID

*NO LO HAN DADO DE ALTA APENAS EN SEPTIEMBRE

*TODAVIA NO LA DAN DE ALTA SIGUE EN CUARENTENA

*DOS DIAS POSITIVO

*SIGUE ENFERMO

**Agregar a "weightloss"

*BAJO DE PESO

*PERDIÓ PESO

*Agregar en "depre"

*EMOCIONALES

**Agregar en "vomit_diarrhea"

*DIARREA

**Agregar en "hyporexia"

*PERDIDA DE APETITO

*

** ¿Lo integro a alguna categoría, como dyspnoea or chest_pain?

*AFCTO SU PULMON

*DOLOR EN LOS PULMONES

*DOLOR EN PULMONES

*iNFLAMACION EN PULMONES

** Agrego una nueva "odinogafia"?

*ARDOR DE GARGANTA

*ARDOR EN GARGANTA

*DOLOR DE GARGANTA

*DOLOR DR GARGANTA

*DOLOR EN GARGANTA

** Los dejo en Otros?

*ALTA Y BAJA DE PRESION

*BAJA DE PRESION

*HIPERTENCION

*PRECION BAJA

*

*ARRIDMIA CARDIACA

*RITMO CARDIACO ELEVADO

*

*BAJA DE PLAQUETAS

*

*CAIDA DE CABELLO

*CAIDA DE PELO

*CAIDA PELO

*PERDIDA DE CABELLO

*PERDIDA DE CABELLO

*PÉRDIDA DE CABELLO

*PERDIDA DE CABELLO Y DOLOR DE ESPALDA

*ESTRÉS Y PÉRDIDA PELO

*

*CONVULSIONES

*

*DAÑO EN LA VISTA

*DISMINUCION DE VISTA

*PROBLEMAS DE LA VISTA

*PROBLEMAS DE VISION

*Dificultad PARA Ver

*

*DESORGANISADA

*

*DOLOR DE ESTOMACAL

*ÚLCERA EN EL ESTOMAGO

*

*ESTORNUDO

*

*FLEMA

*FLEMAS

*

*GANANCIA DE PESO

*

*MANCHAS Y COMEZON EN EL CUERPO

*

*MANO ADORMECIDA

*

*NAUSEAS

*

*OIDOS TAPADOS

*

*PIEL MANCHADA

*SE ESCAMO LA PIEL

*

*SANGRADO

*SANGRADO INTERNO

*

*SUDOR Y BOCHORNO

*SUDORACION

*

*TAQUICARDIA Y PIE CON BARICES O VENA..

*

*TEMBLORES ANTES DE COMER

*TEMBLORINA

*

*TIENES MUCHA SED

*

*VERTIGO

* Generate Symptoms/Sequelae variable (as in the Ensanut questionnaire)

egen sum_sym=rowtotal(cough fatigue anxiety depre fever insomnia renal hyporexia weightloss headache dizziness myalgia_arthralgia dyspnoea chest_pain vomit_diarrhea anosmia ageusia trouble_concen others)

gen symptoms_seque=.

replace symptoms_seque=0 if asymptomatic==1

replace symptoms_seque=1 if sum_sym>0

replace symptoms_seque=2 if less_month==1

label define symptoms_seque 0 "Asymptomatic" 1 "Symptomatic" 2 "Still COVID"

label value symptoms_seque symptoms_seque

tab symptoms_seque

label var symptoms_seque "Symptoms/Sequelae"

* Generate a new day and year variable (se decidió colocar el primero de cada mes)

gen month_COVID=.

replace month_COVID=1 if h1207==1

replace month_COVID=2 if h1207==2

replace month_COVID=3 if h1207==3

replace month_COVID=4 if h1207==4

replace month_COVID=5 if h1207==5

replace month_COVID=6 if h1207==6

replace month_COVID=7 if h1207==7

replace month_COVID=8 if h1207==8

replace month_COVID=9 if h1207==9

replace month_COVID=10 if h1207==10

replace month_COVID=11 if h1207==11

replace month_COVID=12 if h1207==12

label var month_COVID "Date of COVID"

* Generate difference in months

gen months_difference = month_interv-month_COVID

* generate Post acute-COVID and Chronic-COVID variable

gen long_chro=.

replace long_chro=0 if h1215==1 | h1215==2

replace long_chro=1 if h1215==3 | h1215==4

replace long_chro=0 if h1214==1 & months_difference<=3

replace long_chro=1 if h1214==1 & months_difference>3 & months_difference!=.

label define long_chro 0 "Post acute-COVID" 1 "Chronic-COVID"

label value long_chro long_chro

tab long_chro

label var long_chro "Post acute-COVID and Chronic-COVID"

* Review participants with less than 1 month of diagnosis according to self-report

br months_difference month_COVID month_interv symptoms_seque long_chro h1214 h1215 h1205 if months_difference==0 & symptoms_seque==1

br months_difference month_COVID month_interv symptoms_seque long_chro h1214 h1215 h1205 if months_difference<=0 & symptoms_seque==1

* Generate Social security variable

gen derechohabiencia=.

replace derechohabiencia=1 if H0310A==1 //IMSS

replace derechohabiencia=1 if H0310A==07 //IMSS Bienestar

replace derechohabiencia=2 if H0310A==02 //ISSSTE

replace derechohabiencia=2 if H0310A==03 //ISSSTE Estatal

replace derechohabiencia=3 if H0310A==04 //PEMEX

replace derechohabiencia=3 if H0310A==05 //Defensa

replace derechohabiencia=3 if H0310A==06 //Marina

replace derechohabiencia=3 if H0310A==09 //Otra institución: Centro Medico (1 persona)

replace derechohabiencia=4 if H0310A==08 //Privado

replace derechohabiencia=5 if H0310A==10 //No está afiliado (INSABI)

replace derechohabiencia=5 if H0310A==11 //Tenía SP en 2018

replace derechohabiencia=6 if H0310A==99 //NS/NR

label define derechohabiencia 1"IMSS" 2"ISSSTE" 3"Otros públicos" 4"Privado" 5"Sin afiliación" 6"NS/NR"

label values derechohabiencia derechohabiencia

replace derechohabiencia=5 if derechohabiencia==6

*

gen derecho_dico=derechohabiencia

replace derecho_dico=1 if derechohabiencia==1 | derechohabiencia==2 | derechohabiencia==3 | derechohabiencia==4

replace derecho_dico=2 if derechohabiencia==5

label define derecho_dico 1 "With affiliation" 2 "Without affiliation"

label values derecho_dico derecho_dico

* Chronic diseases

gen enf_cronicas=h0902

*

gen diabetes = regexm(enf_cronicas, "1")

gen obesidad = regexm(enf_cronicas, "2")

gen hipertension = regexm(enf_cronicas, "3")

* Total prevalence (post acute COVID/chronic COVID) NICE reference

gen seque=long_chro

recode seque 0=1 1=2

replace seque=0 if symptoms_seque==0

label define seque 0"No sequelae" 1"Post acute-COVID" 2"Chronic-COVID"

label values seque seque

*** New Post-COVID condition variables

* OMS reference (categories: without Post-COVID conditions and Post-COVID conditions (>12 weeks))

gen long_COVID_all=seque

replace long_COVID_all=0 if seque==0 | seque==1

replace long_COVID_all=1 if seque==2

label define long_COVID_all 0 "No sequelae_Ongoing-COVID" 1 "PCC"

label values long_COVID_all long_COVID_all

* NICE reference (categories: without Long-term effects of COVID-19 and Post-COVID-19 syndrome (>12 weeks))

gen long_COVID=.

replace long_COVID=0 if seque==0

replace long_COVID=1 if seque==2

label define long_COVID 0 "No sequelae" 1 "PCS"

label values long_COVID long_COVID

* NICE reference (categories: without Long-term effects of COVID-19 and Ongoing symptomatic COVID-19 (4-12 weeks))

gen long_COVID_4_12=.

replace long_COVID_4_12=0 if seque==0

replace long_COVID_4_12=1 if seque==1

label define long_COVID_4_12 0 "No sequelae" 1 "Long COVID 4-12 weeks"

label values long_COVID_4_12 long_COVID_4_12

*

* Determine sample size

gen ex_age=(h0303==999)

gen ex_child=(h0303<20)

gen ex_nsCO=(h1205==9)

gen ex_noCO=(h1205==2)

gen ex_stillCO=(symptoms_seque==2)

gen ex_edu=(edu_4==.)

gen ex_0month=(months_difference<=0)

egen pre_ex=rowtotal(ex_age-ex_0month)

gen ex_nsmonth=(pre_ex==0 & months_difference==.)

egen total_ex=rowtotal(ex_age-ex_nsmonth)

* Determine sample size (Complete case analysis: exclusions with more than 3 months without COVID diagnosis)

gen ex_3month=(months_difference<=3)

egen pre_ex3=rowtotal(ex_age-ex_edu ex_3month)

gen ex_nsmonth_3=(pre_ex3==0 & months_difference==.)

egen total_ex3=rowtotal(ex_age-ex_edu ex_3month pre_ex3 ex_nsmonth_3)

*** Sensitivity and specificity analysis of chronic diseases

*Diabetes diagnosis

gen diabetes_adul=a0301

recode diabetes_adul 2=.

recode diabetes_adul 3=0

tab diabetes diabetes_adul if total_ex3==0, col miss

tab diabetes_adul

tab diabetes

*

gen hipertension_adul=a0401

recode hipertension_adul 2=.

recode hipertension_adul 3=0

tab hipertension hipertension_adul if total_ex3==0, col miss

tab hipertension_adul

tab hipertension

*

*** New variables that indicates time with diabetes and hypertension diagnosis

* Diabetes

gen time_diabetes= h0303-a0302

replace time_diabetes=. if a0302==99

*

gen diabetes_after_COVID=.

replace diabetes_after_COVID=0 if time_diabetes>0 & time_diabetes!=.

*(1,504 real changes made)

replace diabetes_after_COVID=1 if time_diabetes<=0

*(100 real changes made)

replace diabetes_after_COVID=. if a0301==2

*(19 real changes made, 19 to missing)

label define diabetes_after_COVID 0 "Diabetes before COVID" 1 "Diabetes after COVID"

label values diabetes_after_COVID diabetes_after_COVID

*

* Hypertension

gen time_months_hta=a0402m

replace time_months_hta=. if a0402m==88

replace time_months_hta=. if a0402m==99

*

gen time_years_hta=a0402a

replace time_years_hta=. if a0402a==88

replace time_years_hta=. if a0402a==99

*

gen time_years_to_months_hta=time_years_hta*12

*

gen time_hta=time_months_hta+time_years_to_months_hta

replace time_hta=time_years_to_months_hta if a0402m==99

*(54 real changes made)

replace time_hta=time_years_to_months_hta if a0402m==88

*(4 real changes made)

replace time_hta=time_months_hta if a0402a==99

*(0 real changes made)

replace time_hta=time_months_hta if a0402a==88

*(0 real changes made)

replace time_hta=time_months_hta if a0402a==.

*(112 real changes made)

*

gen hta_minus_COVID=time_hta-months_difference

*

gen hta_after_COVID=.

replace hta_after_COVID=0 if hta_minus_COVID>0 & hta_minus_COVID!=.

*(143 real changes made)

replace hta_after_COVID=1 if hta_minus_COVID<=0

*(22 real changes made)

replace hta_after_COVID=0 if time_hta>11 & time_hta!=. & hta_after_COVID==.

*(1,946 real changes made)

replace hta_after_COVID=1 if time_hta<=11 & hta_after_COVID==.

*(323 real changes made)

replace hta_after_COVID=. if a0401==2

*(59 real changes made, 59 to missing)

label define hta_after_COVID 0 "HTA before COVID" 1 "HTA after COVID"

label values hta_after_COVID hta_after_COVID

*

* SVY set

svyset [pweight =ponde_f], strata(est_sel) psu(upm) singleunit(centered)

*

* Keep only with the final sample size of the analysis

keep if total_ex3==0

*Save dataset

save "$bases/COVID19_SEQUELAE_ENSANUT_2021.dta", replace

*NOTE: 347 variables and 869 observations

* unique FOLIO_I: 633

* unique FOLIO_INT: 869

*==============================================================================*

* DESCRIPTIVE ANALYSIS

* Dataset :

*==============================================================================*

use "$bases/COVID19_SEQUELAE_ENSANUT_2021.dta", clear

* Table 1

putexcel set "/Users/Output_June23.xlsx", modify sh("Table1")

local y=1

putexcel B`y'=("No Long COVID") E`y'=("Long COVID")

local y=`y'+1

putexcel B`y'=("%") C`y'=("lower") D`y'=("upper") E`y'=("%") F`y'=("lower") G`y'=("upper") H`y'=("sample n") I`y'=("weighted n")

local y=`y'+1

quie svy, subpop(if total_ex3==0 ): proportion long_COVID_all

matselrc r(table) ns, row(1,5,6) col(1)

quie svy, subpop(if total_ex3==0 ): proportion long_COVID_all

matselrc r(table) pcs, row(1,5,6) col(2)

putexcel B`y'=matrix((ns)'*100) E`y'=matrix((pcs)'*100) H`y'=(e(N_sub)) I`y'=(e(N_subpop)/1000000)

local y=`y'+2

quie svy, subpop(if total_ex3==0 ): proportion long_COVID_all, over(sex)

matselrc r(table) ns, row(1,5,6) col(1,2)

quie svy, subpop(if total_ex3==0 ): proportion long_COVID_all, over(sex)

matselrc r(table) pcs, row(1,5,6) col(3,4)

quie svy, subpop(if total_ex3==0 ): proportion long_COVID_all, over(sex)

matselrc e(_N) sample, row(1) col(1,2)

quie svy, subpop(if total_ex3==0 ): proportion long_COVID_all, over(sex)

matselrc e(_N_subp) weight, row(1) col(1,2)

putexcel B`y'=matrix((ns)'*100) E`y'=matrix((pcs)'*100) H`y'=matrix((sample)') I`y'=matrix((weight)'/1000000)

local y=`y'+3

quie svy, subpop(if total_ex3==0 ): proportion long_COVID_all, over(age_cat)

matselrc r(table) ns, row(1,5,6) col(1,2,3)

quie svy, subpop(if total_ex3==0 ): proportion long_COVID_all, over(age_cat)

matselrc r(table) pcs, row(1,5,6) col(4,5,6)

quie svy, subpop(if total_ex3==0 ): proportion long_COVID_all, over(age_cat)

matselrc e(_N) sample, row(1) col(1,2,3)

quie svy, subpop(if total_ex3==0 ): proportion long_COVID_all, over(age_cat)

matselrc e(_N_subp) weight, row(1) col(1,2,3)

putexcel B`y'=matrix((ns)'*100) E`y'=matrix((pcs)'*100) H`y'=matrix((sample)') I`y'=matrix((weight)'/1000000)

local y=`y'+4

quie svy, subpop(if total_ex3==0 ): proportion long_COVID_all, over(estrato)

matselrc r(table) ns, row(1,5,6) col(1,2,3)

quie svy, subpop(if total_ex3==0 ): proportion long_COVID_all, over(estrato)

matselrc r(table) pcs, row(1,5,6) col(4,5,6)

quie svy, subpop(if total_ex3==0 ): proportion long_COVID_all, over(estrato)

matselrc e(_N) sample, row(1) col(1,2,3)

quie svy, subpop(if total_ex3==0 ): proportion long_COVID_all, over(estrato)

matselrc e(_N_subp) weight, row(1) col(1,2,3)

putexcel B`y'=matrix((ns)'*100) E`y'=matrix((pcs)'*100) H`y'=matrix((sample)') I`y'=matrix((weight)'/1000000)

local y=`y'+4

quie svy, subpop(if total_ex3==0 ): proportion long_COVID_all, over(derecho_dico)

matselrc r(table) ns, row(1,5,6) col(1,2)

quie svy, subpop(if total_ex3==0 ): proportion long_COVID_all, over(derecho_dico)

matselrc r(table) pcs, row(1,5,6) col(3,4)

quie svy, subpop(if total_ex3==0 ): proportion long_COVID_all, over(derecho_dico)

matselrc e(_N) sample, row(1) col(1,2)

quie svy, subpop(if total_ex3==0 ): proportion long_COVID_all, over(derecho_dico)

matselrc e(_N_subp) weight, row(1) col(1,2)

putexcel B`y'=matrix((ns)'*100) E`y'=matrix((pcs)'*100) H`y'=matrix((sample)') I`y'=matrix((weight)'/1000000)

local y=`y'+3

quie svy, subpop(if total_ex3==0 ): proportion long_COVID_all, over(nseF)

matselrc r(table) ns, row(1,5,6) col(1,2,3)

quie svy, subpop(if total_ex3==0 ): proportion long_COVID_all, over(nseF)

matselrc r(table) pcs, row(1,5,6) col(4,5,6)

quie svy, subpop(if total_ex3==0 ): proportion long_COVID_all, over(nseF)

matselrc e(_N) sample, row(1) col(1,2,3)

quie svy, subpop(if total_ex3==0 ): proportion long_COVID_all, over(nseF)

matselrc e(_N_subp) weight, row(1) col(1,2,3)

putexcel B`y'=matrix((ns)'*100) E`y'=matrix((pcs)'*100) H`y'=matrix((sample)') I`y'=matrix((weight)'/1000000)

local y=`y'+4

quie svy, subpop(if total_ex3==0 ): proportion long_COVID_all, over(edu_4 )

matselrc r(table) ns, row(1,5,6) col(1,2,3,4)

quie svy, subpop(if total_ex3==0 ): proportion long_COVID_all, over(edu_4 )

matselrc r(table) pcs, row(1,5,6) col(5,6,7,8)

quie svy, subpop(if total_ex3==0 ): proportion long_COVID_all, over(edu_4 )

matselrc e(_N) sample, row(1) col(1,2,3,4)

quie svy, subpop(if total_ex3==0 ): proportion long_COVID_all, over(edu_4 )

matselrc e(_N_subp) weight, row(1) col(1,2,3,4)

putexcel B`y'=matrix((ns)'*100) E`y'=matrix((pcs)'*100) H`y'=matrix((sample)') I`y'=matrix((weight)'/1000000)

local y=`y'+5

quie svy, subpop(if total_ex3==0 ): proportion long_COVID_all, over(diabetes )

matselrc r(table) ns, row(1,5,6) col(1,2)

quie svy, subpop(if total_ex3==0 ): proportion long_COVID_all, over(diabetes )

matselrc r(table) pcs, row(1,5,6) col(3,4)

quie svy, subpop(if total_ex3==0 ): proportion long_COVID_all, over(diabetes )

matselrc e(_N) sample, row(1) col(1,2)

quie svy, subpop(if total_ex3==0 ): proportion long_COVID_all, over(diabetes )

matselrc e(_N_subp) weight, row(1) col(1,2)

putexcel B`y'=matrix((ns)'*100) E`y'=matrix((pcs)'*100) H`y'=matrix((sample)') I`y'=matrix((weight)'/1000000)

local y=`y'+3

quie svy, subpop(if total_ex3==0 ): proportion long_COVID_all, over(hipertension)

matselrc r(table) ns, row(1,5,6) col(1,2)

quie svy, subpop(if total_ex3==0 ): proportion long_COVID_all, over(hipertension)

matselrc r(table) pcs, row(1,5,6) col(3,4)

quie svy, subpop(if total_ex3==0 ): proportion long_COVID_all, over(hipertension)

matselrc e(_N) sample, row(1) col(1,2)

quie svy, subpop(if total_ex3==0 ): proportion long_COVID_all, over(hipertension)

matselrc e(_N_subp) weight, row(1) col(1,2)

putexcel B`y'=matrix((ns)'*100) E`y'=matrix((pcs)'*100) H`y'=matrix((sample)') I`y'=matrix((weight)'/1000000)

****FIGURE 2/SYMPTOMS - COLUMN

putexcel set "/Users/Output_June23.xlsx", modify sh("Symptoms")

local y=1

putexcel B`y'=("Long COVID")

local y=`y'+1

putexcel B`y'=("%") C`y'=("lower") D`y'=("upper")

local y=`y'+1

quie svy, subpop(if total_ex3==0 & long_COVID_all==1): proportion renal others vomit_diarrhea dizziness fever trouble_concen hyporexia depre chest_pain weightloss ageusia anxiety anosmia insomnia cough headache dyspnoea myalgia_arthralgia fatigue

matselrc r(table) pcs, row(1,5,6) col(2,4,6,8,10,12,14,16,18,20,22,24,26,28,30,32,34,36,38)

putexcel B`y'=matrix((pcs)'*100)

** Supplementary Table 2

putexcel set "/Users/Output_Aug22.xlsx", modify sh("Table1")

local y=1

putexcel B`y'=("No sequelae") E`y'=("Ongoing-COVID") H`y'=("PCS")

local y=`y'+1

putexcel B`y'=("%") C`y'=("lower") D`y'=("upper") E`y'=("%") F`y'=("lower") G`y'=("upper") H`y'=("%") I`y'=("lower") J`y'=("upper") K`y'=("sample n") L`y'=("weighted n")

local y=`y'+1

quie svy, subpop(if total_ex3==0 ): proportion seque

matselrc r(table) ns, row(1,5,6) col(1)

quie svy, subpop(if total_ex3==0 ): proportion seque

matselrc r(table) ong, row(1,5,6) col(2)

quie svy, subpop(if total_ex3==0 ): proportion seque

matselrc r(table) pcs, row(1,5,6) col(3)

putexcel B`y'=matrix((ns)'*100) E`y'=matrix((ong)'*100) H`y'=matrix((pcs)'*100) K`y'=(e(N_sub)) L`y'=(e(N_subpop)/1000000)

local y=`y'+2

quie svy, subpop(if total_ex3==0 ): proportion seque, over(sex)

matselrc r(table) ns, row(1,5,6) col(1,2)

quie svy, subpop(if total_ex3==0 ): proportion seque, over(sex)

matselrc r(table) ong, row(1,5,6) col(3,4)

quie svy, subpop(if total_ex3==0 ): proportion seque, over(sex)

matselrc r(table) pcs, row(1,5,6) col(5,6)

quie svy, subpop(if total_ex3==0 ): proportion seque, over(sex)

matselrc e(_N) sample, row(1) col(1,2)

quie svy, subpop(if total_ex3==0 ): proportion seque, over(sex)

matselrc e(_N_subp) weight, row(1) col(1,2)

putexcel B`y'=matrix((ns)'*100) E`y'=matrix((ong)'*100) H`y'=matrix((pcs)'*100) K`y'=matrix((sample)') L`y'=matrix((weight)'/1000000)

local y=`y'+3

quie svy, subpop(if total_ex3==0 ): proportion seque, over(age_cat)

matselrc r(table) ns, row(1,5,6) col(1,2,3)

quie svy, subpop(if total_ex3==0 ): proportion seque, over(age_cat)

matselrc r(table) ong, row(1,5,6) col(4,5,6)

quie svy, subpop(if total_ex3==0 ): proportion seque, over(age_cat)

matselrc r(table) pcs, row(1,5,6) col(7,8,9)

quie svy, subpop(if total_ex3==0 ): proportion seque, over(age_cat)

matselrc e(_N) sample, row(1) col(1,2,3)

quie svy, subpop(if total_ex3==0 ): proportion seque, over(age_cat)

matselrc e(_N_subp) weight, row(1) col(1,2,3)

putexcel B`y'=matrix((ns)'*100) E`y'=matrix((ong)'*100) H`y'=matrix((pcs)'*100) K`y'=matrix((sample)') L`y'=matrix((weight)'/1000000)

local y=`y'+4

quie svy, subpop(if total_ex3==0 ): proportion seque, over(estrato)

matselrc r(table) ns, row(1,5,6) col(1,2,3)

quie svy, subpop(if total_ex3==0 ): proportion seque, over(estrato)

matselrc r(table) ong, row(1,5,6) col(4,5,6)

quie svy, subpop(if total_ex3==0 ): proportion seque, over(estrato)

matselrc r(table) pcs, row(1,5,6) col(7,8,9)

quie svy, subpop(if total_ex3==0 ): proportion seque, over(estrato)

matselrc e(_N) sample, row(1) col(1,2,3)

quie svy, subpop(if total_ex3==0 ): proportion seque, over(estrato)

matselrc e(_N_subp) weight, row(1) col(1,2,3)

putexcel B`y'=matrix((ns)'*100) E`y'=matrix((ong)'*100) H`y'=matrix((pcs)'*100) K`y'=matrix((sample)') L`y'=matrix((weight)'/1000000)

local y=`y'+4

quie svy, subpop(if total_ex3==0 ): proportion seque, over(derecho_dico)

matselrc r(table) ns, row(1,5,6) col(1,2)

quie svy, subpop(if total_ex3==0 ): proportion seque, over(derecho_dico)

matselrc r(table) ong, row(1,5,6) col(3,4)

quie svy, subpop(if total_ex3==0 ): proportion seque, over(derecho_dico)

matselrc r(table) pcs, row(1,5,6) col(5,6)

quie svy, subpop(if total_ex3==0 ): proportion seque, over(derecho_dico)

matselrc e(_N) sample, row(1) col(1,2)

quie svy, subpop(if total_ex3==0 ): proportion seque, over(derecho_dico)

matselrc e(_N_subp) weight, row(1) col(1,2)

putexcel B`y'=matrix((ns)'*100) E`y'=matrix((ong)'*100) H`y'=matrix((pcs)'*100) K`y'=matrix((sample)') L`y'=matrix((weight)'/1000000)

local y=`y'+3

quie svy, subpop(if total_ex3==0 ): proportion seque, over(nseF)

matselrc r(table) ns, row(1,5,6) col(1,2,3)

quie svy, subpop(if total_ex3==0 ): proportion seque, over(nseF)

matselrc r(table) ong, row(1,5,6) col(4,5,6)

quie svy, subpop(if total_ex3==0 ): proportion seque, over(nseF)

matselrc r(table) pcs, row(1,5,6) col(7,8,9)

quie svy, subpop(if total_ex3==0 ): proportion seque, over(nseF)

matselrc e(_N) sample, row(1) col(1,2,3)

quie svy, subpop(if total_ex3==0 ): proportion seque, over(nseF)

matselrc e(_N_subp) weight, row(1) col(1,2,3)

putexcel B`y'=matrix((ns)'*100) E`y'=matrix((ong)'*100) H`y'=matrix((pcs)'*100) K`y'=matrix((sample)') L`y'=matrix((weight)'/1000000)

local y=`y'+4

quie svy, subpop(if total_ex3==0 ): proportion seque, over(edu_4 )

matselrc r(table) ns, row(1,5,6) col(1,2,3,4)

quie svy, subpop(if total_ex3==0 ): proportion seque, over(edu_4 )

matselrc r(table) ong, row(1,5,6) col(5,6,7,8)

quie svy, subpop(if total_ex3==0 ): proportion seque, over(edu_4 )

matselrc r(table) pcs, row(1,5,6) col(9,10,11,12)

quie svy, subpop(if total_ex3==0 ): proportion seque, over(edu_4 )

matselrc e(_N) sample, row(1) col(1,2,3,4)

quie svy, subpop(if total_ex3==0 ): proportion seque, over(edu_4 )

matselrc e(_N_subp) weight, row(1) col(1,2,3,4)

putexcel B`y'=matrix((ns)'*100) E`y'=matrix((ong)'*100) H`y'=matrix((pcs)'*100) K`y'=matrix((sample)') L`y'=matrix((weight)'/1000000)

local y=`y'+5

quie svy, subpop(if total_ex3==0 ): proportion seque, over(diabetes )

matselrc r(table) ns, row(1,5,6) col(1,2)

quie svy, subpop(if total_ex3==0 ): proportion seque, over(diabetes )

matselrc r(table) ong, row(1,5,6) col(3,4)

quie svy, subpop(if total_ex3==0 ): proportion seque, over(diabetes )

matselrc r(table) pcs, row(1,5,6) col(5,6)

quie svy, subpop(if total_ex3==0 ): proportion seque, over(diabetes )

matselrc e(_N) sample, row(1) col(1,2)

quie svy, subpop(if total_ex3==0 ): proportion seque, over(diabetes )

matselrc e(_N_subp) weight, row(1) col(1,2)

putexcel B`y'=matrix((ns)'*100) E`y'=matrix((ong)'*100) H`y'=matrix((pcs)'*100) K`y'=matrix((sample)') L`y'=matrix((weight)'/1000000)

local y=`y'+3

quie svy, subpop(if total_ex3==0 ): proportion seque, over(hipertension)

matselrc r(table) ns, row(1,5,6) col(1,2)

quie svy, subpop(if total_ex3==0 ): proportion seque, over(hipertension)

matselrc r(table) ong, row(1,5,6) col(3,4)

quie svy, subpop(if total_ex3==0 ): proportion seque, over(hipertension)

matselrc r(table) pcs, row(1,5,6) col(5,6)

quie svy, subpop(if total_ex3==0 ): proportion seque, over(hipertension)

matselrc e(_N) sample, row(1) col(1,2)

quie svy, subpop(if total_ex3==0 ): proportion seque, over(hipertension)

matselrc e(_N_subp) weight, row(1) col(1,2)

putexcel B`y'=matrix((ns)'*100) E`y'=matrix((ong)'*100) H`y'=matrix((pcs)'*100) K`y'=matrix((sample)') L`y'=matrix((weight)'/1000000)

****Supplementary FIGURE 1/SYMPTOMS - COLUMN

putexcel set "/Users/Output_Aug22.xlsx", modify sh("Symptoms")

local y=1

putexcel B`y'=("Ongoing-COVID") E`y'=("PCS")

local y=`y'+1

putexcel B`y'=("%") C`y'=("lower") D`y'=("upper") E`y'=("%") F`y'=("lower") G`y'=("upper")

local y=`y'+1

quie svy, subpop(if total_ex3==0 ): proportion renal others vomit_diarrhea dizziness fever trouble_concen hyporexia depre chest_pain weightloss ageusia anxiety anosmia insomnia cough headache dyspnoea myalgia_arthralgia fatigue , over(seque)

matselrc r(table) ong, row(1,5,6) col(5,11,17,23,29,35,41,47,53,59,65,71,77,83,89,95,101,107,113)

quie svy, subpop(if total_ex3==0 ): proportion renal others vomit_diarrhea dizziness fever trouble_concen hyporexia depre chest_pain weightloss ageusia anxiety anosmia insomnia cough headache dyspnoea myalgia_arthralgia fatigue , over(seque)

matselrc r(table) pcs, row(1,5,6) col(6,12,18,24,30,36,42,48,54,60,66,72,78,84,90,96,102,108,114)

putexcel B`y'=matrix((ong)'*100) E`y'=matrix((pcs)'*100)

*==============================================================================*

* INFERENTIAL ANALYSIS

* Dataset :

*==============================================================================*

use "$bases/COVID19_SEQUELAE_ENSANUT_2021.dta", clear

* Table 2

*** Binomial regression models with a log link

* Unadjusted Models - Main analysis (WHO reference)

* Diabetes

svy: glm long_COVID_all i.diabetes if total_ex3==0, family(binomial) link(log) eform

*

* Hipertension

svy: glm long_COVID_all i.hipertension if total_ex3==0, family(binomial) link(log) eform

* Adjusted Models

* Diabetes

svy: glm long_COVID_all i.diabetes i.sex h0303 if total_ex3==0, family(binomial) link(log) eform

*

* Hipertension

svy: glm long_COVID_all i.hipertension i.sex h0303 if total_ex3==0, family(binomial) link(log) eform

* Supplementary Table 3

* Unadjusted Models - Sensitivity 2 (NICE reference, category: 4-12 weeks)

* Diabetes

svy: glm long_COVID_4_12 i.diabetes if total_ex3==0, family(binomial) link(log) eform

* Hipertension

svy: glm long_COVID_4_12 i.hipertension if total_ex3==0, family(binomial) link(log) eform

* Adjusted Models

* Diabetes

svy: glm long_COVID_4_12 i.diabetes i.sex h0303 if total_ex3==0, family(binomial) link(log) eform

* Hipertension

svy: glm long_COVID_4_12 i.hipertension i.sex h0303 if total_ex3==0, family(binomial) link(log) eform

* Supplementary Table 4

* Unadjusted Models - Sensitivity 3 (NICE reference, category: >12 weeks)

* Diabetes

svy: glm long_COVID i.diabetes if total_ex3==0, family(binomial) link(log) eform

*

* Hipertension

svy: glm long_COVID i.hipertension if total_ex3==0, family(binomial) link(log) eform

* Adjusted Models

* Diabetes

svy: glm long_COVID i.diabetes i.sex h0303 if total_ex3==0, family(binomial) link(log) eform

*

* Hipertension

svy: glm long_COVID i.hipertension i.sex h0303 if total_ex3==0, family(binomial) link(log) eform

| **Supplementary Table 2. Prevalence of Long-term effects of COVID-19 in Mexican adults diagnosed with COVID-19 in 2021. Ensanut 2021** | | | | | | |
| --- | --- | --- | --- | --- | --- | --- |
|  | **Adults diagnosed with COVID-19** | | **Long-term effects of COVID-19** | | | |
|  |  |  | **Ongoing symptomatic COVID-19 (4-12 weeks)^a^** | | **Post-COVID-19 syndrome (> 12 weeks)^b^** | |
|  | **Sample size unweighted No.** | **Weighted**  **No.**  **(millions)^c^** | **%** | **95%CI** | **%** | **95%CI** |
| **Total** | 869 | 2.5 | 31.8 | 27.8 , 36.1 | 37.0 | 32.7 , 41.5 |
| Sex |  |  |  |  |  |  |
| Men | 381 | 1.2 | 35.1 | 28.5 , 42.3 | 31.4 | 25.4 , 38.1 |
| Women | 488 | 1.4 | 29.0 | 24.3 , 34.2 | 41.7 | 36.8 , 46.8 |
| Age (years) |  |  |  |  |  |  |
| 20-39 | 338 | 1.1 | 34.2 | 28.1 , 41.0 | 33.4 | 27.6 , 39.9 |
| 40-59 | 360 | 1.0 | 30.3 | 24.6 , 36.6 | 38.2 | 31.5 , 45.4 |
| 60 and older | 171 | 0.5 | 29.5 | 20.8 , 40.0 | 42.5 | 31.2 , 54.6 |
| Urbanization (population) |  |  |  |  |  |  |
| Rural (<2,500 inhabitants) | 96 | 0.3 | 24.1 | 16.0 , 34.5 | 48.8 | 37.9 , 59.8 |
| Urban (2,500-100,000 inhabitants) | 264 | 0.7 | 39.1 | 31.7 , 47.0 | 34.8 | 26.5 , 44.1 |
| Metropolitan (>100,000 inhabitants) | 509 | 1.6 | 29.8 | 24.8 , 35.3 | 36.0 | 30.6 , 41.7 |
| Social security |  |  |  |  |  |  |
| With affiliation | 470 | 1.4 | 31.8 | 26.4 , 37.7 | 34.4 | 28.5 , 40.8 |
| Without affiliation | 399 | 1.1 | 31.8 | 26.2 , 38.1 | 40.4 | 34.5 , 46.5 |
| Socioeconomic level |  |  |  |  |  |  |
| Low | 162 | 0.4 | 26.3 | 18.1 , 36.5 | 49.9 | 40.0 , 59.8 |
| Medium | 296 | 0.8 | 31.0 | 24.8 , 38.0 | 36.9 | 29.9 , 44.6 |
| High | 411 | 1.3 | 34.0 | 27.3 , 41.5 | 33.0 | 26.2 , 40.5 |
| Education |  |  |  |  |  |  |
| Elementary school | 189 | 0.5 | 36.6 | 28.0 , 46.1 | 33.1 | 25.4 , 41.8 |
| Middle school | 225 | 0.6 | 32.0 | 25.4 , 39.4 | 42.0 | 34.7 , 49.6 |
| High school | 251 | 0.8 | 27.8 | 22.5 , 33.9 | 39.7 | 32.0 , 48.1 |
| University or higher | 204 | 0.7 | 33.1 | 25.7 , 41.6 | 32.2 | 24.2 , 41.3 |
| Comorbidities |  |  |  |  |  |  |
| Diabetes |  |  |  |  |  |  |
| No | 745 | 2.2 | 31.2 | 27.2 , 35.4 | 35.8 | 31.6 , 40.2 |
| Yes | 124 | 0.4 | 35.5 | 23.5 , 49.7 | 43.7 | 30.5 , 57.9 |
| Hypertension |  |  |  |  |  |  |
| No | 689 | 2.1 | 32.3 | 28.0 , 37.0 | 34.5 | 30.1 , 39.1 |
| Yes | 180 | 0.5 | 29.5 | 21.7 , 38.7 | 47.7 | 37.4 , 58.2 |
| ^a^ Ongoing symptomatic COVID-19 (4-12 weeks): Participants with self-reported positive COVID-19 with presence of signs and symptoms from 4 weeks up to 12 weeks  ^b^ Post-COVID-19 syndrome (> 12 weeks): Participants with self-reported positive COVID-19 with presence of signs and symptoms more than 12 weeks  ^c^ N millions represents the expanded sample size of adults diagnosed with COVID-19. | | | | | | |

**Supplementary Figure 1. Prevalence of symptoms by Long-term effects of COVID-19 duration in Mexican adults diagnosed with COVID-19 in 2021. Ensanut 2021**

Respiratory symptoms include respiratory distress and dyspnea.

| **Supplementary Table 3. Association between diabetes and hypertension with Ongoing symptomatic COVID-19 (4-12 weeks) in Mexican adults diagnosed with COVID-19 in 2021. Ensanut 2021.** | | | |
| --- | --- | --- | --- |
|  | **Ongoing symptomatic COVID-19  (4-12 weeks)^a^** | | |
|  | **Yes vs no** | | |
|  | **Prevalence ratio** | **95% CI** | ***P* value** |
| **Diabetes^b^** |  |  |  |
| **No** | 1 [Reference] | NA | NA |
| **Yes** | 1.34 | 0.94 , 1.89 | 0.103 |
| **Hypertension^b^** |  |  |  |
| **No** | 1 [Reference] | NA | NA |
| **Yes** | 1.14 | 0.92 , 1.40 | 0.237 |
| Abbreviations: 95% CI, 95% Confidence Interval; NA, not applicable. | | | |
| Number of observations = 545 | | | |
| ^a^ Log-binomial regression models adjusted for sex and age. Participants without Post-COVID conditions was used as reference. | | | |
| ^b^Chronic diseases were reported by the head of the household for all family members. | | | |

| **Supplementary Table 4. Association between diabetes and hypertension with Post-COVID-19 syndrome (>12 weeks) in Mexican adults diagnosed with COVID-19 in 2021. Ensanut 2021.** | | | |
| --- | --- | --- | --- |
|  | **Post-COVID-19 syndrome (>12 weeks)l^a^** | | |
|  | **Yes vs no** | | |
|  | **Prevalence ratio** | **95%CI** | ***P* value** |
| **Diabetes^b^** |  |  |  |
| **No** | 1 [Reference] | NA | NA |
| **Yes** | 1.19 | 0.95 , 1.49 | 0.127 |
| **Hypertension^b^** |  |  |  |
| **No** | 1 [Reference] | NA | NA |
| **Yes** | 1.25 | 1.02 , 1.55 | **0.035** |
| Abbreviations: 95% CI, 95% Confidence Interval; NA, not applicable. | | | |
| Number of observations = 601 | | | |
| ^a^Log-binomial regression models adjusted for sex and age. Participants without Post-COVID conditions was used as reference. | | | |
| ^b^Chronic diseases were reported by the head of the household for all family members. | | | |

| **Supplementary Table 5. Concept of the Sensitivity and Specificity screening examinations** | | | |
| --- | --- | --- | --- |
|  |  | **True characteristics in the population** | |
|  |  | **Disease** | **No Disease** |
| **Test results** | **Positive** | True positive (TP) = Have disease and have positive test | False positive (FP) = No disease, but have positive test |
|  | **Negative** | False negative (FN) = Have disease, but have negative test | True negative (TN) = No disease and have negative test |
|  |  |  |  |
|  |  | **Sensitivity = TP / TP+FN** | **Specificity = TN / TN+FP** |

| **Supplementary Table 6. Sensitivity and Specificity of diabetes diagnosis. Ensanut 2021** | | | | |
| --- | --- | --- | --- | --- |
|  |  | **Diabetes diagnosis reported by each adult in the individual health questionnaire (self-reported)** | |  |
|  |  | **Disease** | **No Disease** | Total |
| **Diabetes diagnosis reported by the head of the household in the household questionnaire** | **Positive** | 51 | 8 | 59 |
|  | **Negative** | 3 | 346 | 349 |
|  | Total | 54 | 354 | 408 |
|  |  |  |  |  |
|  |  | **Sensitivity = 51 / (51+3) = 94.4%** | **Specificity = 346 / (346+8) = 97.7%** |  |

| **Supplementary Table 7. Sensitivity and Specificity of hypertension diagnosis. Ensanut 2021** | | | | |
| --- | --- | --- | --- | --- |
|  |  | **Hypertension diagnosis reported by each adult in the individual health questionnaire (self-reported)** | |  |
|  |  | **Disease** | **No Disease** | Total |
| **Hypertension diagnosis reported by the head of the household in the household questionnaire** | **Positive** | 74 | 11 | 85 |
|  | **Negative** | 15 | 308 | 323 |
|  | Total | 89 | 319 | 408 |
|  |  |  |  |  |
|  |  | **Sensitivity = 74 / (74+15) = 83.1%** | **Specificity = 308 / (308+11) = 96.6%** |  |
